# Sequence-Dependent Analgesic Efficacy of Ketamine and Magnesium Sulfate After Radical Nephrectomy

**DOI:** 10.3390/medicina62040754

**Published:** 2026-04-15

**Authors:** Nikola N. Ladjevic, Zoran Dzamic, Vesna D. Jovanovic, Natasa Dj. Petrovic, Svetlana D. Sreckovic, Milos M. Lazic, Branka Terzic, Ivana Likic Ladjevic, Nebojsa Ladjevic

**Affiliations:** 1 Clinic for Urology, University Clinical Center of Serbia, Resavska 51 St., 11000 Belgrade, Serbia; nikola.ladjevic@yahoo.com (N.N.L.); dzamiczoran960@gmail.com (Z.D.); dencic.natasha@gmail.com (N.D.P.); svetlanasreckovic@yahoo.com (S.D.S.); 2 Faculty of Medicine, University of Belgrade, Dr Subotica 8 St., 11000 Belgrade, Serbia; vantonijevicjov@gmail.com (V.D.J.); ivanalikicladjevic@gmail.com (I.L.L.); 3 Center for Anesthesiology and Resuscitation, University Clinical Center of Serbia, Pasterova 2 St., 11000 Belgrade, Serbia; m.lazic1003@gmail.com (M.M.L.); terzic.branka@gmail.com (B.T.); 4 Clinic for Gynecology and Obstetrics, University Clinical Center of Serbia, Koste Todorovića 26, Pasterova 2 St., 11000 Belgrade, Serbia

**Keywords:** ketamine, magnesium sulfate, NMDA receptor antagonist, postoperative pain, radical nephrectomy, sequence-dependent analgesia, multimodal analgesia, central sensitization

## Abstract

*Background and Objectives*: Ketamine and magnesium sulfate (MgSO_4_) are NMDA receptor antagonists that act through distinct mechanisms. Preclinical data indicate that their analgesic interaction is sequence-dependent: ketamine administered before MgSO_4_ produces synergistic antinociception, whereas the reversed sequence is antagonistic. The primary outcomes were postoperative pain intensity (Numerical Rating Scale, NRS 0–10, at rest and on movement) and cumulative intravenous morphine consumption over 48 h, evaluated in patients undergoing open radical nephrectomy to test the hypothesis of sequence-dependent analgesic interaction. *Materials and Methods*: In this randomized, double-blind, placebo-controlled trial, 208 patients scheduled for elective open radical nephrectomy received two sequential intravenous boluses intraoperatively: Drug A immediately after induction, Drug B 10 min later. Agents were ketamine 0.2 mg/kg, MgSO_4_ 15 mg/kg, or placebo (0.9% NaCl) in all nine possible combinations. Primary outcomes were postoperative pain intensity (NRS 0–10, at rest and on movement) and cumulative intravenous morphine consumption, assessed at 14 time points over 48 h. Secondary outcomes included sedation, nausea, vomiting, and the presence of hallucinations. *Results*: The ketamine → MgSO_4_ (K → Mg) sequence significantly reduced NRS pain scores compared to placebo at multiple time points, including 30 min, 1 h, 3 h, 6 h, and 32 h postoperatively, with differences exceeding the minimum clinically important difference of 2 NRS points at the earliest assessments. The MgSO_4_ → ketamine (Mg → K) sequence did not differ from placebo at any time point. Cumulative morphine consumption was comparable across groups. No hallucinations or psychomimetic events were observed. *Conclusions*: Intraoperative ketamine followed by MgSO_4_ (K → Mg) provides clinically meaningful postoperative analgesia after open radical nephrectomy; the reversed sequence (Mg → K) offers no benefit over placebo. These findings provide the first clinical confirmation of sequence-dependent NMDA receptor antagonism and support the K → Mg protocol as a safe, simple addition to multimodal perioperative analgesia. Trial registration: ISRCTN registry, ISRCTN83633282.

## 1. Introduction

Renal cell carcinoma (RCC) represents the most common malignant tumor of the renal parenchyma in adults and constitutes approximately 2.4% of all cancer diagnoses worldwide. According to the 2020 GLOBOCAN data, approximately 400,000 new cases and 180,000 deaths attributable to RCC were recorded globally, with the highest incidence rates observed in developed countries in Europe and North America [1]. More recent estimates from the 2022 GLOBOCAN registry indexed 434,840 new renal cancer cases, with projections suggesting a 72% increase to nearly 746,000 annual cases by 2050, driven largely by population aging and the rising prevalence of modifiable risk factors, including hypertension, obesity, and smoking [2]. In the majority of patients presenting with localized or locally advanced disease, surgical resection—primarily radical nephrectomy, performed via open or laparoscopic approach—remains the cornerstone of curative treatment [3].

Postoperative pain after radical nephrectomy is clinically significant and can be moderate to severe, particularly in the early postoperative period [4]. Peak pain intensity typically occurs within the first 30 to 60 min after surgery, with high rates of analgesic demand in both open and laparoscopic approaches [4]. Beyond the immediate postoperative period, inadequately managed acute pain is associated with prolonged hospital stay, impaired functional recovery, and an increased risk of transition to chronic postsurgical pain (CPSP)—a well-recognized complication of major abdominal and urological surgery affecting approximately 11–16% of patients at two months after nephrectomy [4]. Effective perioperative analgesic strategies that attenuate peak pain intensity and suppress the neuroplastic changes underlying central sensitization are, therefore, a clinical priority in this surgical population.

Opioids have traditionally formed the backbone of postoperative pain management, but their use is associated with significant adverse effects, including respiratory depression, nausea, vomiting, excessive sedation, ileus, and the risk of dependency—concerns that have grown more prominent in the context of the ongoing opioid crisis [5]. This has driven increasing interest in multimodal, opioid-sparing analgesic strategies that target pain through complementary pharmacological mechanisms, reduce opioid requirements, and improve the overall safety profile of perioperative care [5,6].

Central to the mechanism of postoperative pain hypersensitivity and the development of CPSP is the phenomenon of central sensitization—a state of neuronal hyperexcitability in the spinal dorsal horn induced by sustained nociceptive input from the surgical site. The induction and maintenance of central sensitization are critically dependent on activation of N-methyl-D-aspartate (NMDA) receptors, as established by the foundational work of Woolf and Thompson [7]. NMDA receptor antagonists, by interrupting this sensitization process perioperatively, have been shown to reduce postoperative pain intensity, decrease opioid consumption, and potentially prevent the establishment of chronic pain states [8].

Among NMDA receptor antagonists available for perioperative use, ketamine and magnesium sulfate have attracted particular interest owing to their distinct yet complementary mechanisms of receptor blockade, well-characterized safety profiles, and low cost. Ketamine is a non-competitive open-channel blocker that inhibits NMDA receptors during periods of nociceptive neuronal activation. It has been extensively studied in perioperative pain management and shown in multiple systematic reviews and meta-analyses to reduce postoperative pain scores and opioid consumption across a wide range of surgical procedures [8,9]. Magnesium sulfate is an endogenous voltage-dependent channel blocker of the NMDA receptor; it occupies the channel pore at resting membrane potentials and inhibits receptor opening in a physiologically relevant manner [10]. Although its analgesic efficacy when used alone has been inconsistent across clinical trials, multiple umbrella reviews and meta-analyses have demonstrated a significant overall benefit on postoperative pain and analgesic consumption when magnesium is administered perioperatively [11].

The combination of ketamine and magnesium sulfate is pharmacologically compelling because the two agents block the NMDA receptor through mechanistically distinct pathways and have been shown to interact in a super-additive (synergistic) manner at the molecular level. Liu et al. demonstrated in a series of in vitro studies using Xenopus oocytes that the co-application of ketamine and Mg^2+^ significantly decreased the half-maximal inhibitory concentration of each agent—by more than 90%—compared to either agent alone, with isobolographic analysis confirming super-additive interaction [12]. This molecular synergism provides a strong preclinical rationale for the clinical use of the combination in perioperative analgesia.

However, preclinical pharmacological data have also revealed that the direction of the pharmacodynamic interaction between ketamine and magnesium is not fixed but critically depends on the sequence of administration. The literature demonstrated in the rat tail-immersion model that, while individual administration of either agent at low doses produced no significant antinociception, their combination produced synergistic inhibition of acute nociception—but only at specific dose ratios and with ketamine administered before magnesium [13]. The same group subsequently showed, in a rat formalin-induced inflammatory pain model, that when ketamine was administered after magnesium, the interaction became antagonistic rather than synergistic, requiring approximately 1.6-fold higher ketamine doses to achieve the same analgesic effect [14]. Furthermore, in a morphine-ketamine-magnesium triple combination, the antinociceptive efficacy was substantially reduced when magnesium preceded ketamine but was enhanced when the reverse sequence was used [15]. These preclinical findings constitute a specific, experimentally verified hypothesis: that, in clinical practice, the sequence K → Mg (ketamine first, then magnesium) should produce superior analgesia compared with Mg → K, and that the latter may confer no benefit over placebo.

Radical nephrectomy is a particularly compelling surgical model for investigating combinations of NMDA receptor antagonists for several reasons. First, patients undergoing this procedure carry a high prevalence of hypertension, obesity, and impaired renal reserve—comorbidities that influence the pharmacokinetics of both ketamine and magnesium sulfate. Although ketamine is primarily hepatically metabolised, renal dysfunction reduces its clearance by approximately 20% and leads to accumulation of its active metabolite dehydronorketamine [16,17]. Renal excretion is the primary route of magnesium elimination, with over 60% of an intravenous dose excreted renally under normal conditions [18]; the anticipated reduction in glomerular filtration rate following unilateral nephrectomy thus introduces a clinically relevant pharmacokinetic consideration that is absent in most other surgical models. Second, the pain profile of open radical nephrectomy is characterised by a large flank incision involving the 11th and 12th intercostal nerves, extensive paraspinal muscle division, peritoneal retraction, and potential diaphragmatic irritation, generating a high-intensity, prolonged nociceptive input that is both somatic and visceral in nature [19,20]—conditions particularly favourable for evaluating the antinociceptive efficacy of NMDA receptor blockade. Third, unlike elective procedures performed in otherwise healthy patients, radical nephrectomy is performed in an oncological context, where minimising perioperative opioid exposure carries additional relevance given emerging evidence linking opioid-induced immunosuppression to impaired anti-tumour immunity and a potential increased risk of cancer recurrence [21,22]. Despite its clinical relevance as a surgical model with a specific pain profile and pharmacologically relevant patient population, the sequence-dependent interaction between ketamine and magnesium has never been directly tested in patients undergoing radical nephrectomy.

Despite this mechanistic rationale, the sequence-dependent interaction between ketamine and magnesium has never been directly tested in a prospective clinical trial. Published studies on the ketamine-magnesium combination in the perioperative setting have either administered the agents simultaneously [23], or used unspecified sequences [24,25], precluding any evaluation of whether sequence affects clinical outcomes. Moreover, no prior study has investigated this combination in patients undergoing radical nephrectomy—a procedure characterized by a specific pain profile, a patient population with a high prevalence of hypertension and metabolic comorbidities, and a documented risk of CPSP.

The present study was therefore designed as a randomized, double-blind, placebo-controlled trial with a primary objective of evaluating the sequence-dependent analgesic efficacy of intraoperative ketamine and magnesium sulfate in patients undergoing radical nephrectomy. Patients were allocated to one of nine study groups defined by the identity and sequence of two sequential intraoperative boluses: Each of the three possible agents—ketamine 0.2 mg/kg, MgSO_4_ 15 mg/kg, or placebo (0.9% NaCl)—could occupy either the Drug A or Drug B position, yielding nine subgroups: Ketamine → MgSO_4_ (K → Mg), MgSO_4_ → Ketamine (Mg → K), Ketamine → Ketamine (K → K), MgSO_4_ → MgSO_4_ (Mg → Mg), Ketamine → Placebo (K → Pl), Placebo → Ketamine (Pl → K), MgSO_4_ → Placebo (Mg → Pl), Placebo → MgSO_4_ (Pl → Mg), and Placebo → Placebo (Pl → Pl). The primary comparisons of interest were between the K → Mg, Mg → K, and Pl → Pl groups The primary outcomes were postoperative pain scores at rest and on movement measured by the Numerical Rating Scale (NRS) at multiple time points over 48 h, and postoperative morphine consumption. Secondary objectives included assessment of psychomimetic adverse effects, sedation, and hemodynamic parameters.

We hypothesized that the K → Mg sequence would produce significantly lower postoperative pain scores than placebo and the reversed Mg → K sequence, based on preclinical evidence of sequence-dependent synergism. We further hypothesized that the Mg → K sequence would not differ significantly from placebo, based on the preclinical evidence of antagonistic interaction when magnesium precedes ketamine. The present report describes the primary clinical outcomes of this trial.

## 2. Materials and Methods

### 2.1. Study Design and Setting

This was a randomized, double-blind, placebo-controlled clinical trial evaluating the sequence-dependent analgesic efficacy of perioperative intravenous ketamine and magnesium sulfate (MgSO_4_) in patients undergoing elective open radical nephrectomy. The study was conducted at the Department of Urology and the Centre for Anesthesiology and Resuscitation, University Clinical Centre of Serbia, Belgrade, Serbia, between August 2017 and January 2026. The study was approved by the Institutional Ethics Committee of the University Clinical Centre of Serbia (approval number: 361/15, date: 13 July 2017) and was conducted in accordance with the Declaration of Helsinki. The trial was retrospective registered in the ISRCTN registry (ISRCTN83633282); the completed CONSORT 2025 checklist is provided as Supplementary Table S1. Written informed consent was obtained from all participants prior to enrollment.

### 2.2. Participants

A total of 208 patients were enrolled. Eligible participants were adults (≥18 years of age) with a unilateral renal tumor for whom radical nephrectomy had been indicated by the responsible urologist. The following inclusion and exclusion criteria were applied:

Inclusion criteria: American Society of Anesthesiologists (ASA) physical status class I–III; age ≥ 18 years; unilateral renal tumor scheduled for open radical nephrectomy.

Exclusion criteria: resting bradycardia or atrioventricular (AV) block; New York Heart Association (NYHA) functional class > 2; glomerular filtration rate (GFR) < 50 mL/min; severe or poorly controlled chronic obstructive pulmonary disease (COPD) or asthma; poorly controlled arterial hypertension; severe hepatic impairment; elevated intraocular or intracranial pressure (IOP or ICP); significant psychiatric comorbidity (excluding mild anxiety or mild depression); known allergy to any of the study medications; pre-existing chronic pain syndrome; or regular use of analgesic medications in the month preceding surgery.

### 2.3. Randomization and Blinding

Patients were randomized into one of nine study subgroups defined by the identity and sequence of two intraoperative bolus injections (Drug A administered immediately after induction of anesthesia, Drug B administered 10 min later, at least 10 min before surgical incision). Each of the three possible agents—ketamine 0.2 mg/kg, MgSO_4_ 15 mg/kg, or placebo (0.9% NaCl)—could occupy either the Drug A or Drug B position, yielding the following nine subgroups: Ketamine → MgSO_4_ (K → Mg), MgSO_4_ → Ketamine (Mg → K), Ketamine → Ketamine (K → K), MgSO_4_ → MgSO_4_ (Mg → Mg), Ketamine → Placebo (K → Pl), Placebo → Ketamine (Pl → K), MgSO_4_ → Placebo (Mg → Pl), Placebo → MgSO_4_ (Pl → Mg), and Placebo → Placebo (Pl → Pl).

All three study solutions were prepared by an anesthesia nurse not involved in patient care or data collection, in identical syringes of equal volume, and were visually indistinguishable (colorless, transparent solutions). The attending anesthesiologist, surgical team, and all postoperative assessors were blinded to group allocation throughout the study. Patients were not informed of their treatment assignment; because all three study solutions were prepared in identical, colorless, visually indistinguishable syringes of equal volume, patients had no means of identifying the administered agents. Postoperative pain assessments were performed by nursing staff independent of the anesthetic team, who had no access to the randomization list. The blinding of the anesthesiologist was maintained by having the study solutions labeled only with a patient code and delivered directly to the operating room by the independent preparation nurse immediately before administration. The randomization sequence was generated prior to study commencement using a computer-based random number generator with block randomization (SPSS v26.0, IBM Corp.) and was maintained by a designated independent coordinator not involved in patient care, recruitment, or outcome assessment. The allocation sequence was stored in sequentially numbered, sealed, opaque envelopes held exclusively by the independent coordinator and disclosed only immediately prior to the preparation of study solutions for each enrolled patient. The attending anesthesiologist and all clinical personnel had no access to the allocation list at any time. Block sizes were varied randomly to preserve allocation concealment; as a result, given the sequential enrollment over an extended study period, the final per-group sample sizes were not constrained to be equal. The observed variation in group size (ranging from 17 to 37 participants) is an expected consequence of this design and does not reflect differential weighting of any particular treatment sequence.

### 2.4. Preoperative Assessment

Prior to surgery, the following data were collected from all participants: sex, age, body weight, body height, and body mass index (BMI); smoking status; current comorbidities; history of prior renal or other surgical procedures; laterality of the tumor (left or right kidney); and patient-anticipated pain intensity assessed via the Numerical Rating Scale (NRS) as a preoperative pain expectation measure.

### 2.5. Anesthetic Protocol

All patients received the same standardized premedication administered intramuscularly approximately 30 min before transfer to the operating room: midazolam 0.04 mg/kg and atropine 0.5 mg. General anesthesia was induced identically in all participants with intravenous fentanyl 2 μg/kg, propofol 2 mg/kg, and rocuronium 0.6 mg/kg. Tracheal intubation was performed in all cases. Anesthesia was maintained with sevoflurane (MAC = 1.0) in a 40% oxygen–60% air mixture, supplemented with intraoperative fentanyl and rocuronium as required based on the duration and course of the surgery.

The study interventions (Drug A and Drug B) were administered as intravenous boluses according to the following standardized sequence: Drug A was given immediately after induction of anesthesia; Drug B was given 10 min after Drug A, and at least 10 min before surgical incision. All patients received intravenous paracetamol 1 g administered 30 min before emergence from anesthesia.

### 2.6. Intraoperative Data Collection

The following intraoperative parameters were recorded for each patient: duration of surgery (min); intraoperative blood loss (mL); total dose of intraoperative fentanyl administered (mL; 1 mL = 0.05 mg); and the occurrence of intraoperative events such as pleural injury or rib resection.

### 2.7. Postoperative Analgesic Protocol

On admission to the postoperative care unit (PACU) or intensive care unit (ICU), all patients received a standardized multimodal analgesic regimen. Paracetamol 1 g intravenously was administered every 8 h as scheduled baseline analgesia. Rescue analgesia was provided with intravenous morphine administered in 1 mg incremental boluses, repeatable every 10 min, until the patient reported pain relief to a level below NRS ≤ 3 at rest (static pain) or NRS < 5 on movement, deep breathing, or cough (dynamic pain). Total morphine consumption (mg) and the time to first morphine administration were recorded in the case report form (CRF) for each patient.

### 2.8. Outcome Measures

The primary outcome was postoperative pain intensity, assessed using the Numerical Rating Scale (NRS; 0–10: 0 = no pain, 1–3 = mild, 4–6 = moderate, ≥7 = severe), at rest and with movement. The secondary primary outcome was cumulative postoperative morphine consumption (mg). Both outcomes were assessed at the following standardized time points after admission to the PACU/ICU: 0 (arrival), 30 min, 1, 2, 3, 4, 6, 8, 12, 16, 20, 24, 32, and 48 h postoperatively.

Additional secondary outcomes recorded at each assessment time point included: Pain intensity assessed by the Verbal Rating Scale (VRS: 0 = no pain, 1 = mild, 2 = moderate, 3 = moderately severe, 4 = severe, 5 = unbearable) at rest and on movement; Nausea severity (0 = none, 1 = mild, 2 = moderate, 3 = significant, 4 = severe); Vomiting (presence/absence) and requirement for metoclopramide rescue; Presence of hallucinations (yes/no); Level of sedation assessed on a 5-point scale (0 = alert and awake; 1 = drowsy; 2 = sleepy but arousable; 3 = responds to mechanical stimulation only; 4 = unresponsive to mechanical stimulation); Postoperative bleeding (presence/extent).

### 2.9. Statistical Analysis

This was a prospective, randomized, double-blind, placebo-controlled trial with nine parallel treatment arms. The primary outcome of this trial was postoperative pain intensity, assessed by the Numerical Rating Scale (NRS, 0–10) at rest and on movement at 14 standardized time points over 48 h; cumulative postoperative morphine consumption over 48 h constituted the secondary outcome. Sample size was calculated a priori based on the primary outcome, using the NRS score at 1 h postoperatively as the reference time point for power calculation. Assuming a clinically meaningful difference of 1.5 NRS points between groups, a standard deviation of 2.5, a two-sided significance level of α = 0.05, and a desired power of 80%, a minimum of 17 patients per group was required. To account for potential dropouts and increase statistical power, enrollment was expanded to 208 patients, distributed across 9 groups (17–37 per group).

Continuous variables with approximately normal distribution are presented as mean ± standard deviation (Mean ± SD). Continuous variables with non-normal distributions—including all pain scores and morphine consumption data—are presented as medians with interquartile ranges (median, IQR: Q1–Q3). Categorical variables are expressed as absolute frequencies and percentages, *n* (%). The normality of continuous variables was assessed using the Shapiro–Wilk test, given the relatively small sample sizes within individual groups. Variables that significantly deviated from normality (*p* < 0.05) were analyzed using non-parametric methods throughout. Baseline demographic and clinical characteristics were compared across all nine study groups to confirm successful randomization. Continuous variables were compared using one-way analysis of variance (ANOVA) where normality assumptions were met, or the Kruskal–Wallis test where they were not. Categorical variables were compared using Pearson’s Chi-square test or Fisher’s exact test, depending on whether expected cell counts were less than 5.

The primary outcome—postoperative pain intensity at rest and on movement, measured by NRS (0–10) and Verbal Rating Scale (VRS, 0–5)—was assessed at 14 standardized time points: wake-up (0), 30 min, 1, 2, 3, 4, 6, 8, 12, 16, 20, 24, 32, and 48 h postoperatively. Given the non-normal distribution of pain scores and the multiple-group design, overall between-group differences at each time point were evaluated using the Kruskal–Wallis test. Planned pairwise comparisons between the three key groups of interest—K → Mg (*n* = 32), Mg → K (*n* = 19), and Pl → Pl (*n* = 37)—were performed using the Mann–Whitney U test (two-sided). These comparisons were pre-specified based on the study hypothesis regarding sequence-dependent drug effects and served as the primary inferential analysis.

Secondary outcomes included total postoperative morphine consumption (number of patient-controlled analgesia boluses over 48 h) and time to first analgesic request (minutes from end of surgery). These were compared across groups using the Kruskal–Wallis test, with Mann–Whitney U used for pairwise comparisons where indicated.

The incidence of adverse events—including nausea and vomiting (assessed at each time point), excessive sedation (Ramsay Sedation Scale score > 1), hallucinations, and emergence excitation—was compared between groups using the Chi-square test or Fisher’s exact test as appropriate. Cumulative nausea incidence over the first 24 h was calculated as the proportion of patients with at least one nausea episode at any time point in that window.

To assess the independent analgesic contribution of magnesium sulfate as a pharmacological class, all groups receiving MgSO_4_ in any sequence (*n* = 95) were pooled and compared against groups receiving no magnesium (*n* = 113) at selected time points using the Mann–Whitney U test. This analysis was exploratory and hypothesis-generating. The relationship between patient age, body weight, BMI, duration of surgery, intraoperative fentanyl dose, and preoperative pain expectation (assessed via NRS prior to surgery) with actual postoperative pain at 1 h was evaluated using Spearman’s rank correlation coefficient. The convergent validity and consistency of the two pain assessment instruments—NRS (numerical) and VRS (verbal)—were examined using Spearman’s rank correlation at each postoperative time point across the full study cohort. Given the exploratory nature of the pairwise comparisons across nine groups and multiple time points, no formal correction for multiple comparisons (e.g., the Bonferroni correction) was applied, in accordance with current practice for pilot and exploratory clinical trials. All reported *p*-values are unadjusted. Findings should be interpreted in the context of the overall pattern of results rather than as isolated individual tests.

All randomized patients (*n* = 208) were included in the primary analysis. There were no protocol deviations requiring exclusion, no withdrawals after randomization, and no missing primary outcome data; the per-protocol population was therefore identical to the intention-to-treat (ITT) population. All analyses were thus conducted on the ITT population.

All statistical analyses were performed using IBM SPSS Statistics, version 26.0 (IBM Corp., Armonk, NY, USA). Figures were generated using Python 3.12 (matplotlib library, version 3.8). A two-sided *p*-value of <0.05 was considered statistically significant throughout.

## 3. Results

### 3.1. Baseline Characteristics

A total of 208 patients undergoing elective radical nephrectomy were enrolled and randomized into nine treatment groups (Table 1); the flow of participants through all stages of the trial is presented in Supplementary Figure S1. Group sizes ranged from 17 to 37 patients, reflecting the unequal allocation inherent in the factorial design. No statistically significant differences were observed between groups with respect to age (*p* = 0.793), body weight (*p* = 0.369), BMI (*p* = 0.258), duration of surgery (*p* = 0.463), intraoperative fentanyl dose (*p* = 0.730), sex distribution (*p* = 0.287), or smoking status (*p* = 0.177), confirming successful randomization and baseline comparability across all study arms. The prevalence of arterial hypertension (61.5%) and other comorbidities (68.8%) was consistent with the expected profile of patients undergoing radical nephrectomy for renal cell carcinoma, and did not differ significantly between groups. ASA physical status classification was predominantly ASA I–II (93.3%), indicating a relatively homogeneous perioperative risk profile.

### 3.2. Postoperative Pain at Rest

**Figure 1:**
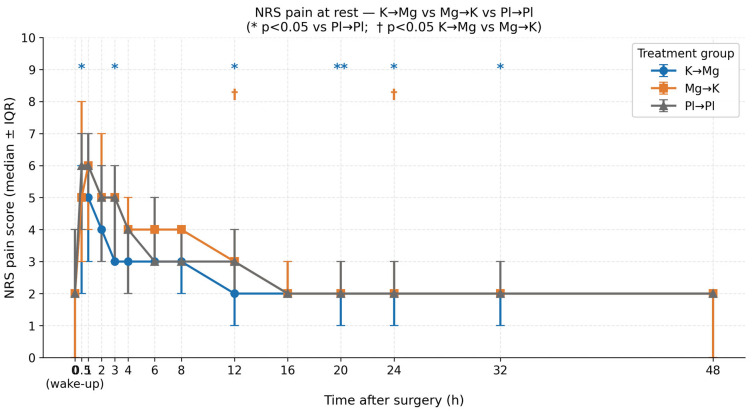
NRS pain scores at rest over 48 h postoperatively (median ± IQR). Asterisks (*) indicate statistically significant differences between K → Mg and Pl → Pl (*p* < 0.05, Mann–Whitney U). Daggers (†) indicate significant differences between K → Mg and Mg → K (*p* < 0.05). Double asterisks (**) indicate significant differences between K→Mg and both Mg→K and Pl→Pl (*p* < 0.05).

NRS pain scores at rest were assessed at 14 standardized time points over 48 h. Overall, between-group comparisons using the Kruskal–Wallis test were not statistically significant at any time point (all *p* > 0.05), as expected given the nine-group design and moderate per-group sample sizes. The primary inferential analysis therefore focused on pre-specified pairwise comparisons between the three key groups: K → Mg (*n* = 32), Mg → K (*n* = 19), and Pl → Pl (*n* = 37). These comparisons revealed a consistent, clinically meaningful pattern of analgesic superiority for the K → Mg regimen that persisted throughout the 32 h observation window (Table 2, Figure 1).

In the early postoperative period, K → Mg produced significantly lower pain scores compared to Pl → Pl at 30 min (median 5.0 vs. 6.0; *p* = 0.032) and at 3 h (median 3.0 vs. 5.0; *p* = 0.014). At 1 h, the difference approached but did not reach statistical significance (median 5.0 vs. 6.0; *p* = 0.067), suggesting a clinically relevant trend during this period. From 3 h onward, the K → Mg group showed a more rapid decline in pain scores than both the Mg → K and Pl → Pl groups, reaching a median NRS of 3 by 3 h, whereas the comparator groups remained at a median NRS of 5.

This analgesic advantage was sustained and, importantly, became more pronounced during the late postoperative period. K → Mg remained significantly lower than Pl → Pl at 12 h (median 2.0 vs. 3.0; *p* = 0.014), 20 h (median 2.0 vs. 2.0; *p* = 0.007), 24 h (median 2.0 vs. 2.0; *p* = 0.035), and 32 h (median 2.0 vs. 2.0; *p* = 0.047). The persistence of this effect beyond 20 h is particularly noteworthy given the relatively short half-lives of both ketamine and magnesium sulfate, and may suggest a sustained modulation of central sensitization rather than a simple pharmacokinetic effect.

The sequence of drug administration proved to be a critical determinant of analgesic efficacy. At both 12 and 24 h postoperatively, K → Mg was statistically superior not only to placebo but also to the reversed sequence Mg → K (12 h: median 2.0 vs. 3.0, *p* = 0.047; 24 h: median 2.0 vs. 2.0, *p* = 0.030). Crucially, no statistically significant differences were observed between Mg → K and Pl → Pl at any time point (all *p* > 0.05), indicating that reversing the sequence of administration—giving magnesium sulfate before ketamine—effectively abolishes the analgesic benefit of the combination and reduces its efficacy to a level indistinguishable from placebo. This finding strongly supports the hypothesis that priming with ketamine is a prerequisite for the full expression of the analgesic synergy between the two agents.

At 48 h, pain scores had converged across all three groups with no significant differences remaining, suggesting that the pharmacological benefit of K → Mg is primarily confined to the first 32 h of the postoperative period.

### 3.3. Postoperative Pain on Movement

**Figure 2:**
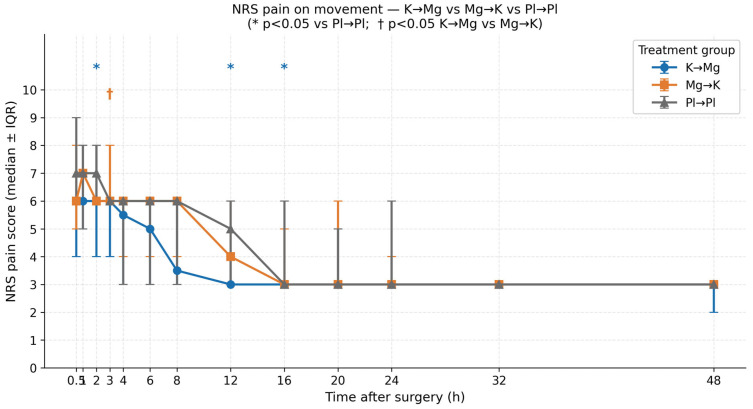
NRS pain scores on movement over 48 h postoperatively (median ± IQR). Asterisks (*) indicate statistically significant differences between K → Mg and Pl → Pl (*p* < 0.05). Daggers (†) indicate significant differences between K → Mg and Mg → K (*p* < 0.05).

Dynamic pain scores were consistently higher than resting scores across all groups and time points, as expected given the physiological nature of postoperative incisional pain. Nevertheless, the K → Mg group demonstrated significantly lower NRS scores for movement than placebo at multiple time points, following a pattern broadly consistent with the findings for resting pain (Table 3, Figure 2).

In the early postoperative period, pain on movement was high across all groups, reflecting the immediate postoperative state. At 30 min, K → Mg showed a numerically lower score than Pl → Pl (median 6.0 vs. 7.0), but this difference did not reach statistical significance (*p* = 0.080). By 2 h, the difference was statistically significant (median 6.0 vs. 7.0; *p* = 0.016), suggesting that the analgesic effect of the K → Mg combination, although not immediately apparent during dynamic activity, becomes clinically evident within the first 2 postoperative hours.

In the late postoperative period, K → Mg continued to demonstrate superior pain control on movement compared to placebo at 12 h (median 3.0 vs. 5.0; *p* = 0.025) and 16 h (median 3.0 vs. 3.0; *p* = 0.023). The difference at 12 h was particularly clinically meaningful, representing a two-point reduction in NRS—a threshold widely considered the minimum clinically important difference for pain on a numerical scale.

As with resting pain, the sequence of administration influenced analgesic outcomes for dynamic pain. At 3 h postoperatively, K → Mg produced significantly lower pain on movement than Mg → K (median 6.0 vs. 6.0; *p* = 0.041), despite identical numerical medians, reflecting a difference in the distribution of scores within the groups. At 8 h, a similar trend was observed (median 3.5 vs. 6.0; *p* = 0.091), approaching but not reaching statistical significance. Consistent with the resting pain findings, no significant differences were observed between Mg → K and Pl → Pl for dynamic pain at any time point, further reinforcing the conclusion that the analgesic effect is sequence-dependent rather than simply additive when the two drugs are administered independently.

Beyond 24 h, pain scores on movement converged across all groups, with all groups reaching a median NRS of 3 by 32 h, indicating that the window of differential analgesic effect for dynamic pain is concentrated in the first 24 postoperative hours.

### 3.4. Morphine Consumption

**Figure 3:**
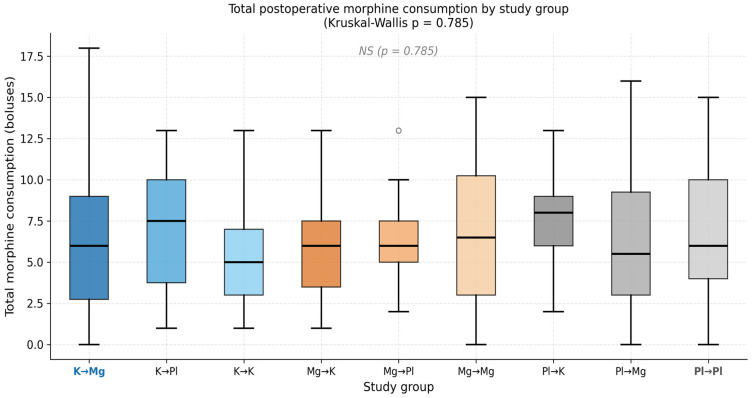
Total postoperative morphine consumption (boluses) by study group. Box plots show median, IQR, and range. Kruskal–Wallis *p* = 0.785 across all nine groups.

Total postoperative morphine consumption, recorded as the number of patient-controlled analgesia boluses over 48 h, did not differ significantly across the nine study groups (Kruskal–Wallis *p* = 0.785). In the three key comparison groups, median total morphine was 6 boluses (IQR 3–9) in K → Mg, 6 boluses (IQR 4–8) in Mg → K, and 6 boluses (IQR 4–10) in Pl → Pl (Table 4, Figure 3). The identical medians and overlapping IQRs across groups indicate that analgesic rescue demand was uniformly distributed regardless of treatment allocation.

Time to first analgesic request was similarly comparable across groups: 25 min (IQR 19–40) in K → Mg, 25 min (IQR 20–60) in Mg → K, and 20 min (IQR 10–50) in Pl → Pl (*p* = 0.248). Although K → Mg patients had a numerically longer median time to first request than Pl → Pl (25 vs. 20 min), this difference was not statistically significant. The wide IQRs in all groups reflect the well-known inter-individual variability in postoperative analgesic requirements and the influence of patient-specific factors such as pain tolerance, preoperative expectations, and intraoperative management.

The absence of a significant reduction in morphine consumption despite demonstrably lower reported pain intensity in the K → Mg group at multiple time points warrants careful interpretation. This apparent discrepancy is most likely explained by the protocol-driven nature of PCA delivery: all patients had equal, unrestricted access to rescue analgesia regardless of group assignment, and the PCA bolus protocol was standardized across groups. Under these conditions, patients may request morphine at a similar absolute threshold of pain regardless of their baseline pain level, effectively equalizing morphine consumption across groups. An alternative interpretation is that the analgesic benefit of K → Mg manifests as a qualitative reduction in pain experience—reducing peak pain intensity and duration—rather than as a quantitative reduction in opioid demand detectable under standard PCA conditions. Future studies employing continuous pain measurement or opioid-sparing endpoints may be better positioned to detect morphine-sparing effects of this combination.

## 4. Discussion

This randomized, double-blind, placebo-controlled trial is, to our knowledge, the first study to systematically investigate the analgesic effect of sequence-dependent sequential administration of ketamine and magnesium sulfate in patients undergoing radical nephrectomy. Our principal finding is that the K → Mg regimen—ketamine 0.2 mg/kg administered intraoperatively, followed by magnesium sulfate 15 mg/kg—produced statistically and clinically significant reductions in postoperative pain scores at rest and with movement compared with placebo at multiple time points over 32 h. Critically, the analgesic benefit was sequence-dependent: the reversed protocol (Mg → K) did not differ from placebo at any time point, suggesting that the order of drug administration—not merely the combination itself—is a prerequisite for achieving the observed analgesic effect. Morphine consumption was similar across all groups, and no hallucinations or psychomimetic excitation were recorded in any patient.

Radical nephrectomy, whether performed via open or laparoscopic approach, is associated with significant postoperative pain that peaks in the early postoperative period and may persist for 24 to 48 h. Alper and Yüksel [4], in a prospective trial of 52 patients undergoing laparoscopic or open radical nephrectomy for renal cell carcinoma, demonstrated that the highest pain scores were recorded at 30 min and 1 h after surgery in both groups, and that over 88–96% of patients required additional analgesia in the early postoperative period. Importantly, their study also showed that patients undergoing laparoscopic and open nephrectomy carried equal risk for developing chronic postsurgical pain (CPSP), with an incidence of approximately 11–16% at 2 months. These findings support the need for effective perioperative analgesic strategies that reduce peak pain intensity and potentially prevent CPSP.

In our study, pain scores in the placebo group were highest at 30 min (median NRS 6.0 at rest, 7.0 on movement) and at 1 h, consistent with the temporal pattern described by Alper and Yüksel [4] and with broader data on postoperative nephrectomy pain. Furthermore, Dillenburg et al. demonstrated, in a comparative analysis of retroperitoneoscopic versus open radical nephrectomy, that postoperative pain and quality of life were significantly compromised regardless of surgical approach, underscoring that analgesic optimization is a surgical priority [26]. The K → Mg sequence reduced this early peak substantially, reaching a median NRS of 5.0 versus 6.0 at 30 min and 3.0 versus 5.0 at 3 h—effects that are not only statistically significant but clinically meaningful, given that a 2-point reduction on the NRS is widely recognized as the minimum clinically important difference [26].

The clinical utility of the K → Mg regimen is supported by the magnitude of the observed NRS reductions: at the 30 min, 1 h, and 3 h postoperative time points, differences between K → Mg and placebo exceeded the widely accepted minimum clinically important difference (MCID) of 2 NRS points, confirming that the statistically significant findings translate into a clinically meaningful analgesic benefit for the patient. Both ketamine and magnesium sulfate act as antagonists of the N-methyl-D-aspartate (NMDA) receptor, albeit through distinct and complementary mechanisms. Ketamine is a non-competitive open-channel blocker that inhibits activated NMDA receptors by binding within the ion channel pore when the channel is open [12]. Magnesium, in contrast, is an endogenous voltage-dependent blocker that occupies the NMDA receptor channel at resting membrane potentials, maintaining a physiological block that prevents channel opening unless sufficient membrane depolarization occurs [27,28].

The induction and maintenance of central sensitization—the process by which spinal dorsal horn neurons become hyperexcitable following peripheral tissue injury—is critically dependent on NMDA receptor activation, as first demonstrated by Woolf and Thompson in their foundational 1991 study [29]. This central sensitization, mediated by temporal summation (wind-up) of C-fiber inputs, is the neurophysiological substrate underlying postoperative hyperalgesia and is increasingly recognized as a driver of the transition from acute to chronic postsurgical pain [30]. NMDA antagonists interrupt this process by preventing the activation cascade that leads to increased intracellular calcium influx, activation of protein kinase C, and long-term potentiation of nociceptive synapses [31].

The interaction between ketamine and magnesium at the NMDA receptor has been studied at the molecular level. Liu et al. [12] demonstrated in Xenopus oocytes expressing NR1/NR2A and NR1/NR2B glutamate receptors that ketamine and magnesium ions act in a super-additive (synergistic) manner when combined—their combined inhibitory effect on NMDA receptor function exceeds the sum of their individual effects. This provides a compelling molecular basis for the clinical expectation that the combination should outperform either agent alone.

The sequence effect observed in our study, however, cannot be explained simply by this synergism. The critical and novel finding is that K → Mg was clinically effective while Mg → K was not. This is consistent with preclinical data from Savić Vujović et al. [13], who demonstrated in the rat tail-immersion model that, while magnesium and ketamine, when given individually, produced no significant antinociception, their combination produced synergistic inhibition of acute nociception—but only at specific dose ratios and sequences. Critically, the same group subsequently showed, in a formalin inflammatory pain model [14], that when ketamine was given after magnesium sulfate, the interaction became antagonistic—prior magnesium administration increased the ketamine dose required to achieve analgesia by approximately 1.6-fold. The same authors further demonstrated that in a morphine-ketamine-magnesium triple combination, analgesic efficacy was markedly reduced when magnesium preceded ketamine, but enhanced when magnesium followed ketamine [14].

The mechanistic explanation for this sequence dependence is biologically plausible. Magnesium ions, when administered first, may occupy and stabilize the NMDA channel in its closed, voltage-dependent blocked state at resting membrane potentials. When ketamine is then administered, it requires an open-channel state to exert its blocking effect—a state that magnesium pretreatment makes more difficult to achieve, as the voltage-dependent Mg^2+^ block prevents the initial depolarizations necessary for channel opening. In other words, Mg^2+^ priming may reduce the NMDA receptor’s susceptibility to ketamine’s open-channel block. Conversely, when ketamine is administered first and blocks the channel in its open state during periods of nociceptive input, subsequent magnesium may reinforce this closed-channel block and prolong the duration of receptor inhibition by preventing re-opening—a scenario consistent with the sustained analgesic effect we observed extending to 32 h.

This sequence-dependent pharmacodynamics is also supported by the clinical observations of Varas et al. [23], who found that intraoperative co-administration of ketamine and magnesium reduced morphine consumption in patients undergoing abdominoplasty, and those of Jabbour et al. [24], who demonstrated in a randomized double-blind trial of scoliosis surgery that sequential ketamine-magnesium combination reduced cumulative morphine consumption by approximately 30% compared to ketamine alone. The present study extends these observations by specifically testing sequence order and focusing on a distinct urological surgical population.

The analgesic efficacy of low-dose perioperative ketamine is well-established across a variety of surgical settings. Brinck et al. [8], in their Cochrane systematic review of perioperative intravenous ketamine for acute postoperative pain in adults, concluded that ketamine significantly reduces postoperative pain intensity and opioid consumption, whether given as an intraoperative bolus alone or followed by a postoperative infusion. Edinoff et al. [9], in a comprehensive narrative review, emphasized that the analgesic effect of low-dose ketamine is most consistently demonstrated for procedures associated with moderate-to-severe postoperative pain, particularly in abdominal, orthopedic, and spinal surgery.

Wang et al. [32], in a systematic review and meta-analysis of S-ketamine for acute postoperative pain including 905 patients from multiple RCTs, found that intravenous S-ketamine significantly decreased pain intensity in the early postoperative period and reduced opioid requirements, but noted a higher incidence of psychotomimetic adverse events compared to placebo. The dose of ketamine used in the present study—0.2 mg/kg—falls at the lower end of the subanesthetic analgesic range. The rationale for this dose was to minimize psychomimetic adverse effects while preserving analgesic synergy with magnesium sulfate.

Remérand et al. [33] demonstrated in a randomized trial of total hip arthroplasty that the analgesic effects of ketamine persist substantially beyond its plasma half-life, suggesting a mechanism involving sustained neuroplastic changes rather than simple receptor occupancy during the dosing period—an interpretation consistent with our finding of significant analgesic benefit at 20–32 h after a single intraoperative bolus. Sun et al. [34], in a meta-analysis of 20 RCTs including 1561 patients, found that perioperative intravenous ketamine significantly reduced the risk of chronic postsurgical pain at 3–6 months postoperatively (RR 0.86, 95% CI 0.77–0.95), suggesting that the benefit of ketamine extends beyond the immediate postoperative period. While our study was not designed to assess chronic pain outcomes, this evidence supports the hypothesis that the K → Mg sequence may carry long-term protective potential in patients undergoing radical nephrectomy.

The analgesic properties of perioperative magnesium sulfate have been extensively studied since the first randomized trial by Tramèr et al. in 1996 [28]. Since then, multiple systematic reviews and meta-analyses have evaluated its efficacy with variable conclusions, largely reflecting heterogeneity in dose, route, timing, and surgical context. Albrecht et al. [35], in an umbrella review of systematic reviews and an updated meta-analysis of RCTs, concluded that perioperative magnesium administration significantly improves postoperative analgesia across a broad range of surgical procedures, identifying NMDA receptor antagonism and calcium channel blockade as its dual mechanistic pillars.

Peng and Huang [36], in a systematic review of 11 RCTs in orthopedic surgery, found that magnesium sulfate reduced postoperative pain intensity in 55% of trials and significantly reduced analgesic consumption in 73%. Avci et al. [37], in a more recent meta-analysis focused on general abdominal surgery, similarly found meaningful reductions in NRS at both early and late timepoints. Given this evidence, the finding that Mg → K was not significantly different from placebo in our study is not attributable to a general lack of magnesium efficacy, but rather to the sequence-specific pharmacodynamic antagonism.

The importance of magnesium dosing and administration protocol should also be noted. In studies demonstrating the most robust analgesic effects, magnesium was administered as a bolus followed by continuous infusion, or at higher doses [36,37]. In our study, a single bolus of 15 mg/kg was used without subsequent infusion. While this is clinically practical, it may limit the duration of the magnesium effect when given as the initial agent, further contributing to the inferior performance of the Mg → K sequence.

The molecular synergism between ketamine and magnesium at NMDA receptors, established by Liu et al. [12], has been further explored in several preclinical studies. Savić Vujović et al. [13] demonstrated in the rat tail-immersion model that specific ketamine-magnesium combinations produced synergistic effects not observed with either agent alone, but also highlighted the dose- and sequence-dependent nature of this interaction. Their subsequent formalin model data [14] confirmed that the pharmacodynamic direction—synergistic versus antagonistic—is sequence-dependent, providing the most direct preclinical support for our clinical findings.

In clinical studies, the K + Mg combination has been investigated in several surgical contexts with mostly favorable results when the two drugs are given simultaneously or ketamine first. Jabbour et al. [24] found in scoliosis surgery that ketamine-magnesium co-administration reduced cumulative morphine consumption significantly compared to ketamine alone, with benefits observed from postoperative hour 4 through hour 48. Hassan et al. [25], in a randomized, double-blind trial of breast cancer surgery, found that ketamine plus magnesium infusion reduced intraoperative analgesic requirements and reduced total postoperative opioid consumption compared with ketamine alone, attributing this to superadditive modulation of NMDA receptors. Conversely, Adhikary et al. [38] found no analgesic advantage of combined bolus ketamine and magnesium after laparoscopic sleeve gastrectomy—a result that may, in retrospect, be explained by sequence-related antagonism or insufficient dosing in a specific bariatric population with altered pharmacokinetics.

The absence of a statistically significant difference in postoperative morphine consumption between K → Mg, Mg → K, and Pl → Pl groups (*p* = 0.785) requires interpretation in the context of significantly lower pain scores in the K → Mg group. This apparent paradox—lower pain intensity without reduced opioid demand—has been observed in other ketamine and magnesium trials and reflects important limitations of PCA-based opioid measurement.

The most parsimonious explanation relates to the standardized design of the PCA protocol. Because all patients had equal, unrestricted access to morphine boluses, the analgesic benefit of K → Mg likely manifested qualitatively—as a reduction in the subjective pain experience at equivalent morphine doses—rather than quantitatively as a measurable reduction in opioid demand. Patients in all groups activated their PCA at a similar perceived pain threshold, but K → Mg patients experienced that threshold at a genuinely lower NRS level. Jabbour et al. [24] observed a significant morphine-sparing effect only when magnesium was continuously infused alongside ketamine, not with a single bolus protocol, suggesting that the opioid-sparing effect of bolus-only protocols may require more sensitive detection methods.

Varas et al. [23] also noted that the morphine reduction with ketamine-magnesium combination was detectable only through multiple regression analysis in some subgroups. The numerically longer time to first analgesic request in K → Mg patients (25 min vs. 20 min in Pl → Pl) hints at a genuine, if non-statistically significant, delay in opioid demand. Future studies employing fixed-interval opioid recording, continuous pain monitoring, or larger sample sizes may better characterize the opioid-sparing potential of this combination.

A key concern with perioperative ketamine administration is the potential for psychomimetic adverse effects, including hallucinations, dysphoria, dissociation, and emergence excitation. In the present study, zero hallucinations and zero psychomimetic excitation were recorded across all 208 patients over the 12 h observation period—a highly clinically significant finding that directly supports the safety of the 0.2 mg/kg ketamine dose in this patient population.

The dose of ketamine used falls within the range defined as subanesthetic, below the threshold at which psychomimetic effects are commonly clinically observed. Schwenk et al. [39], in their consensus guidelines from ASRA, AAPM, and ASA, define subanesthetic dosing as a bolus of ≤0.35 mg/kg or an infusion of ≤1 mg/kg/h, and specify that this range carries a clinically acceptable psychomimetic risk profile. Gorlin et al. [40] reviewed the literature on intravenous subanesthetic ketamine and reported that infusion rates of 0.12–0.2 mg/kg/h produce no increase in psychomimetic events compared to placebo, and that bolus doses below 0.5 mg/kg are generally well tolerated without major psychiatric disturbances.

The meta-analysis by Wang et al. [32] reported a higher incidence of psychotomimetic adverse events with S-ketamine than with placebo; however, many of the included studies used higher doses or longer infusion durations than the current study. The zero-incidence finding in our cohort of 208 patients is particularly robust given the large sample size. This observation is consistent with data from Edinoff et al. [9] and Sun et al. [34], both of whom noted that psychomimetic effects are dose-dependent and clinically rare below 0.3 mg/kg. Excessive sedation was recorded in 5–9% of patients in the key comparison groups, within the range expected in patients receiving PCA morphine and not exceeding placebo rates. This safety profile strongly supports the clinical utility of the K → Mg regimen in routine perioperative practice, with no additional monitoring required.

The primary conceptual contribution of this study is the demonstration that the order of administration of two pharmacologically related NMDA-acting agents determines whether their combination is clinically beneficial or ineffective. This finding has direct implications for anesthesiologists designing multimodal analgesic protocols: sequencing of NMDA-acting adjuvants is not an interchangeable decision but a clinically meaningful variable.

The practical implication is straightforward and directly actionable: when ketamine and magnesium sulfate are used as part of an intraoperative analgesic protocol, ketamine should be administered before magnesium sulfate. The reversed sequence appears to confer no analgesic benefit over placebo in our data, and preclinical evidence from Savić Vujović et al. [13,14] suggests it may reduce the combination’s efficacy.

This finding also invites reconsideration of previously published trials that used simultaneous or unspecified-sequence administration of these agents. If the pharmacodynamic interaction is truly sequence-dependent, trials that did not standardize or report the order of administration may have observed attenuated effects in some patients due to the antagonistic potential of Mg → K. This could partially explain the heterogeneity observed in meta-analyses of perioperative magnesium [35,36] and might represent a resolvable source of inter-study variability that future systematic reviews should explicitly address.

Our results should be interpreted within the framework of multimodal analgesia, which is the current standard of care for perioperative pain management [6]. In this context, K → Mg represents a safe, pharmacologically rational, and clinically effective adjunct to standard perioperative protocols. The combination is inexpensive, easy to administer as sequential intraoperative bolus injections, requires no special monitoring beyond routine anesthetic care, and does not increase opioid consumption, sedation, or nausea compared to placebo.

The MgSO_4_ class effect identified in our exploratory analysis—pooled Mg-containing groups vs. non-Mg groups showing significantly lower pain at 3 h (*p* = 0.007)—further indicates that magnesium exerts an independent analgesic contribution to the perioperative protocol regardless of sequence, particularly in the early postoperative period. This is consistent with the broader evidence base reviewed above and confirms that including magnesium sulfate in perioperative analgesic protocols is valuable even beyond the specific K → Mg sequence.

The findings of the present study are directly relevant to contemporary perioperative care frameworks, particularly Enhanced Recovery After Surgery (ERAS) protocols, which identify opioid minimization as a central pillar of postoperative management [41]. ERAS guidelines for urological and major abdominal surgery explicitly recommend multimodal analgesia incorporating non-opioid adjuncts—including NMDA receptor antagonists—to reduce perioperative opioid consumption and its associated adverse effects [42]. Both ketamine and magnesium sulfate fulfill the pharmacological criteria for opioid-sparing adjuncts outlined in these guidelines, and the K → Mg sequence identified in the present trial provides a practical, low-cost implementation that requires no specialized monitoring or postoperative continuation. Of note, the concept of sequence-dependent pharmacodynamic effects is not unique to the ketamine–magnesium interaction: analogous sequence-dependent phenomena have been described for other NMDA-acting agents, and the temporal order of receptor priming is increasingly recognized as a clinically actionable variable in multimodal analgesia [13,14,15]. The present data add the first prospective clinical evidence that this pharmacological principle translates directly into differential patient outcomes, establishing sequence as a dimension of drug administration that deserves explicit consideration in future perioperative analgesic protocols and ERAS pathway design.

Several limitations of the present study must be acknowledged. First, the nine-group factorial design yielded moderate per-group sample sizes, reducing statistical power for overall between-group Kruskal–Wallis comparisons. The prespecified pairwise comparisons between K → Mg, Mg → K, and Pl → Pl were adequately powered, but the full nine-group comparisons should be interpreted with caution given the smaller group sizes. Second, only a single intraoperative bolus dose was administered for each agent, without subsequent postoperative infusions. Continuous infusion protocols may produce more pronounced analgesic and opioid-sparing effects, as suggested by Jabbour et al. [24] and the broader ketamine literature [8]. Third, while we assessed hallucinations and psychomimetic excitation with standard clinical tools, more sensitive instruments such as the Clinician-Administered Dissociative States Scale (CADSS) were not employed; subclinical dissociative effects could have been missed. Fourth, chronic postsurgical pain was not assessed. Given the evidence that ketamine reduces CPSP incidence [34] and that nephrectomy carries a non-negligible risk of chronic pain [4], future studies should include long-term follow-up at 3 and 6 months. Fifth, the study was conducted at a single center in a predominantly ASA I–II patient population undergoing open radical nephrectomy; generalizability to laparoscopic approaches and higher-risk patient populations requires further investigation. Sixth, although the primary pairwise comparisons between K → Mg, Mg → K, and Pl → Pl were pre-specified and hypothesis-driven, the analysis of NRS scores across 14 time points constitutes multiple comparisons, which increases the probability of Type I error. The study was designed and pre-specified as exploratory, which justified the omission of Bonferroni or other corrections; however, findings should be interpreted conservatively and confirmatory trials with appropriate multiplicity adjustments will be needed to establish definitive significance thresholds.

Based on the findings of this study, several research priorities emerge. First, a prospective head-to-head trial directly comparing K → Mg with simultaneous K + Mg would clarify whether the superior analgesic efficacy is attributable to the pharmacodynamic sequence per se or to the timing of bolus delivery. Second, dose-finding studies for the magnesium component are needed to optimize the balance between analgesic efficacy and hemodynamic effects at higher doses. Third, mechanistic studies using quantitative sensory testing—cold pressor threshold, temporal summation, pressure pain threshold—would provide objective evidence for the central sensitization-modulating hypothesis. Fourth, pharmacokinetic and pharmacodynamic modeling of the ketamine-magnesium interaction in humans would validate and potentially quantify the sequence-dependent receptor binding patterns suggested by preclinical data. Finally, investigating the K → Mg protocol in laparoscopic nephrectomy cohorts and in patients with preoperative chronic pain would enhance the generalizability of these findings.

## 5. Conclusions

This randomized, double-blind, placebo-controlled trial, for the first time in a clinical setting, investigated whether the sequence of administration of two NMDA receptor antagonists—ketamine and magnesium sulfate—determines the magnitude of postoperative analgesia in patients undergoing open radical nephrectomy. The principal finding is unambiguous: the sequence of administration is a critical determinant of analgesic outcome, and the direction of this effect is consistent with the preclinical pharmacological data that motivated the study design.

The K → Mg regimen—intravenous ketamine 0.2 mg/kg administered immediately after induction of anesthesia, followed 10 min later by MgSO_4_ 15 mg/kg—produced statistically significant and clinically meaningful reductions in postoperative pain intensity compared to placebo at multiple time points spanning the first 32 h after surgery. Pain scores at rest and on movement were reduced by margins that exceed the accepted minimum clinically important difference of 2 NRS points at the most pharmacologically relevant early time points (30 min, 1 h, and 3 h postoperatively). Conversely, the reversed sequence—Mg → K—was indistinguishable from placebo across all assessment points, demonstrating that the order of administration does not merely modulate the magnitude of the effect but fundamentally determines whether any analgesic benefit is present at all.

These clinical findings provide the first prospective human evidence confirming the sequence-dependent pharmacodynamic interaction between ketamine and magnesium, previously described only in preclinical rodent models. The antagonistic interaction observed when magnesium precedes ketamine is mechanistically consistent with the electrophysiological properties of the NMDA receptor channel: magnesium, as a voltage-dependent open-channel blocker acting preferentially at resting membrane potentials, stabilizes the channel in a closed configuration that is inaccessible to the subsequent binding of ketamine, an open-channel blocker that requires an activated, open receptor to exert its effect. When the sequence is reversed, ketamine first occupies open channels during the period of heightened nociceptive neuronal activity surrounding surgical stimulation, and the subsequent administration of magnesium reinforces and prolongs this blockade—yielding the synergistic, sustained analgesia observed in the K → Mg group through 32 h postoperatively.

The safety profile of the K → Mg regimen in this surgical population was excellent. No hallucinations or psychomimetic adverse events were documented in any patient receiving ketamine across the entire cohort, consistent with the established safety of subanesthetic ketamine doses when kept below the psychomimetic threshold. Sedation and hemodynamic parameters were comparable across groups. These findings support the routine perioperative safety of the K → Mg protocol in ASA I–III patients undergoing major urological surgery.

Postoperative morphine consumption did not differ significantly between the K → Mg and placebo groups in this study, a finding likely attributable to the structure of the nurse-administered rescue morphine protocol rather than to a lack of pharmacological effect. Under the protocol used, morphine was titrated to a fixed NRS threshold in all groups; patients in the K → Mg group, who experienced lower baseline pain, received morphine at a lower absolute pain level but not necessarily in smaller total doses, as the activation threshold for rescue was similar across groups. The qualitative improvement in pain control—lower peak and sustained pain intensity—represents a clinically meaningful benefit independent of opioid-sparing, particularly in the context of early postoperative recovery, patient comfort, and the prevention of central sensitization.

From a practical standpoint, the K → Mg protocol is notable for its simplicity, cost-effectiveness, and applicability across diverse surgical settings. Both agents are widely available, inexpensive generic medications with established safety profiles. The intervention requires only two-timed intravenous bolus injections within the first 10–20 min of anesthesia, with no requirement for postoperative infusions, specialized monitoring, or advanced equipment. These characteristics make the K → Mg sequence a readily implementable component of multimodal perioperative analgesic pathways in centers performing radical nephrectomy, particularly where neuraxial or regional analgesic techniques are unavailable or contraindicated.

The present study has several limitations that should be acknowledged. The nine-group factorial design, while providing comprehensive pharmacological coverage of all possible drug-sequence combinations, resulted in moderate per-group sample sizes that may have limited statistical power for secondary outcomes. The use of single intravenous boluses of both agents, without postoperative infusion maintenance, may have attenuated opioid-sparing effects that could be achievable with continuous administration protocols. The absence of psychometric monitoring using validated instruments, such as the Clinician-Administered Dissociative States Scale (CADSS), means that subclinical dissociative effects cannot be ruled out. The study was conducted at a single tertiary referral center with a specific patient population (ASA I–III, open radical nephrectomy), limiting the direct generalizability of the findings to laparoscopic or robotic approaches and higher-risk populations. Finally, the study did not include long-term follow-up to assess the impact of perioperative K → Mg analgesia on the incidence of chronic postsurgical pain at 3 or 6 months.

In conclusion, this trial establishes that ketamine followed by magnesium sulfate (K → Mg), administered as sequential intraoperative boluses, provides clinically significant perioperative analgesia in patients undergoing open radical nephrectomy, while the reversed sequence (Mg → K) confers no analgesic advantage over placebo. These findings translate a specific preclinical pharmacodynamic hypothesis into clinical practice and introduce the concept of sequence-dependent NMDA receptor antagonism as a pharmacologically actionable variable in perioperative pain management. Future research should address the optimal dosing and infusion strategies for the K → Mg combination, its efficacy in minimally invasive nephrectomy approaches, and its potential to reduce the incidence of chronic postsurgical pain in this patient population.

## Figures and Tables

**Table 1:** Demographic and clinical characteristics by study group.

Variable	K → Mg (*n* = 32)	K → Pl (*n* = 20)	K → K (*n* = 20)	Mg → K (*n* = 19)	Mg → Pl (*n* = 19)	Mg → Mg (*n* = 20)	Pl → K (*n* = 17)	Pl → Mg (*n* = 24)	Pl → Pl (*n* = 37)	*p*
Age (year), Mean ± SD	58.3 ± 12.6	57.6 ± 7.4	60.2 ± 10.4	57.9 ± 12.0	58.7 ± 12.9	62.3 ± 10.4	60.7 ± 11.0	56.2 ± 11.9	60.9 ± 10.1	0.793
Body weight (kg), Mean ± SD	86.6 ± 15.4	80.0 ± 13.7	82.8 ± 19.0	78.7 ± 13.0	86.4 ± 11.5	80.5 ± 12.6	76.3 ± 13.5	83.5 ± 15.0	80.2 ± 14.1	0.369
BMI (kg/m^2^), Mean ± SD	28.4 ± 4.6	25.9 ± 3.4	26.0 ± 4.0	26.4 ± 3.7	27.5 ± 2.5	26.4 ± 3.9	26.3 ± 4.3	26.9 ± 3.0	26.2 ± 2.6	0.258
Duration of surgery (min), Mean ± SD	108.8 ± 36.2	109.8 ± 35.0	105.8 ± 33.9	105.3 ± 22.4	107.6 ± 27.0	107.5 ± 13.3	91.2 ± 16.9	104.2 ± 22.2	100.0 ± 26.3	0.463
Fentanyl (mcg), Mean ± SD	409 ± 82.7	377 ± 88.1	392 ± 113.9	400 ± 86.6	402 ± 51.3	395 ± 80.9	379 ± 84.9	385 ± 87.8	423 ± 102.5	0.730
Male sex, *n* (%)	19 (59%)	14 (70%)	12 (60%)	8 (42%)	14 (74%)	13 (65%)	6 (35%)	14 (58%)	19 (51%)	0.287
Smokers, *n* (%)	10 (31%)	7 (35%)	9 (45%)	9 (47%)	7 (37%)	3 (15%)	5 (29%)	11 (46%)	7 (19%)	0.177

Values are Mean ± SD or *n* (%). *p*-values from one-way ANOVA (continuous variables) or Chi-square test (categorical variables). BMI = body mass index.

**Table 2:** Postoperative pain intensity at rest (NRS, 0–10) across measurement timepoints for the K→Mg, Mg→K, and Pl→Pl groups.

Time	K → Mg Median (IQR)	Mg → K Median (IQR)	Pl → Pl Median (IQR)	p K → Mg vs. Mg → K	p K → Mg vs. Pl → Pl	p Mg → K vs. Pl → Pl	Conclusion
Wake-up	2.0 (0–4)	2.0 (0–4)	2.0 (2–4)	0.741	0.459	0.260	NS
30 min	5.0 (2–6)	5.0 (3–8)	6.0 (4–7)	0.365	**0.032**	0.372	K → Mg < Pl → Pl *
1 h	5.0 (3–7)	6.0 (4–7)	6.0 (5–7)	0.157	0.067	0.902	Trend (*p* = 0.067)
2 h	4.0 (3–6)	5.0 (4–7)	5.0 (3–6)	0.108	0.151	0.629	NS
3 h	3.0 (3–5)	5.0 (3–5)	5.0 (3–6)	0.056	**0.014**	0.699	K → Mg < Pl → Pl *
4 h	3.0 (2–4)	4.0 (2–5)	4.0 (2–4)	0.114	0.175	0.403	NS
6 h	3.0 (3–4)	4.0 (3–5)	3.0 (3–5)	0.155	0.374	0.654	NS
8 h	3.0 (2–3)	4.0 (3–4)	3.0 (3–4)	0.138	0.160	0.776	NS
12 h	2.0 (1–3)	3.0 (2–4)	3.0 (2–4)	**0.047**	**0.014**	0.780	K → Mg < both **
20 h	2.0 (1–2)	2.0 (2–3)	2.0 (2–3)	0.070	**0.007**	0.570	K → Mg < Pl → Pl *
24 h	2.0 (1–2)	2.0 (2–3)	2.0 (2–3)	**0.030**	**0.035**	0.786	K → Mg < both **
32 h	2.0 (1–2)	2.0 (2–2)	2.0 (2–3)	0.533	**0.047**	0.154	K → Mg < Pl → Pl *
48 h	2.0 (0–2)	2.0 (0–2)	2.0 (2–2)	0.737	0.103	0.305	NS

Values are median (IQR). Bold *p*-values: *p* < 0.05 (Mann–Whitney U, two-sided). * K → Mg significantly lower than Pl → Pl. ** K → Mg significantly lower than both Mg → K and Pl → Pl. NS = not significant.

**Table 3:** NRS pain scores on movement—pairwise comparisons between K → Mg, Mg → K, and Pl → Pl.

Time	K → Mg Median (IQR)	Mg → K Median (IQR)	Pl → Pl Median (IQR)	p K → Mg vs. Mg → K	p K → Mg vs. Pl → Pl	Conclusion
30 min	6.0 (4–8)	6.0 (5–8)	7.0 (6–9)	0.578	0.080	NS (trend)
1 h	6.0 (5–8)	7.0 (6–8)	7.0 (5–8)	0.375	0.457	NS
2 h	6.0 (4–8)	6.0 (6–8)	7.0 (6–8)	0.116	**0.016**	K → Mg < Pl → Pl *
3 h	6.0 (4–6)	6.0 (6–8)	6.0 (6–6)	**0.041**	0.065	K → Mg < Mg → K *
4 h	5.5 (3–6)	6.0 (4–6)	6.0 (3–6)	0.298	0.346	NS
6 h	5.0 (3–6)	6.0 (4–6)	6.0 (3–6)	0.232	0.156	NS
8 h	3.5 (3–6)	6.0 (4–6)	6.0 (3–6)	0.091	0.072	NS (trend)
12 h	3.0 (3–4)	4.0 (3–6)	5.0 (3–6)	0.229	**0.025**	K → Mg < Pl → Pl *
16 h	3.0 (3–3)	3.0 (3–5)	3.0 (3–6)	0.231	**0.023**	K → Mg < Pl → Pl *
24 h	3.0 (3–4)	3.0 (3–4)	3.0 (3–6)	0.633	0.440	NS
48 h	3.0 (2–3)	3.0 (3–3)	3.0 (3–3)	0.656	0.670	NS

Values are median (IQR). Bold *p*-values: *p* < 0.05 (Mann–Whitney U, two-sided). * Statistically significant difference as indicated. NS = not significant.

**Table 4:** Total morphine consumption and time to first analgesic request—key comparison groups.

Variable	K → Mg (*n* = 32)	Mg → K (*n* = 19)	Pl → Pl (*n* = 37)	*p*-Value (Kruskal–Wallis)
Total morphine (boluses), Median (IQR)	6 (3–9)	6 (4–8)	6 (4–10)	0.785
Time to first dose (min), Median (IQR)	25 (19–40)	25 (20–60)	20 (10–50)	0.248

Values are median (IQR). Kruskal–Wallis test across all nine groups.

## Data Availability

The data that support the findings of this study are available on request from the corresponding author.

## Supplementary Materials

The following supporting information can be downloaded at: https://www.mdpi.com/article/10.3390/medicina62040754/s1, Table S1. CONSORT 2025 checklist for the randomized controlled trial Sequence-Dependent Analgesic Efficacy of Ketamine and Magnesium Sulfate After Radical Nephrectomy [43]. Figure S1. CONSORT flow diagram illustrating the enrollment, randomization, and analysis of participants across all nine treatment arms.
