# Supplementary material for: Sequence-Dependent Analgesic Efficacy of Ketamine and Magnesium Sulfate After Radical Nephrectomy
Source: Medicina (Kaunas). 2026 Apr 15;62(4):754. doi: 10.3390/medicina62040754 (PMC13117051; doi:10.3390/medicina62040754)
Supplement: Supplementary file 1 [file medicina-62-00754-s001.zip › medicina-4217316-supplementary.pdf]

CONSORT 2025 CHECKLIST

Sequence-Dependent Analgesic Efficacy of Ketamine and Magnesium Sulfate After Radical Nephrectomy

Lađević et al. — Submitted to Medicina MDPI

Table S1. CONSORT 2025 checklist for the randomized controlled trial "Sequence-Dependent Analgesic Efficacy of Ketamine and Magnesium Sulfate After Radical Nephrectomy.

Item	Checklist Item (CONSORT 2025)	Location in Manuscript	Remarks
**TITLE AND ABSTRACT**			
1a	Identification as a randomised trial in the title	Title page	Present: 'randomised, double-blind, placebo-controlled trial' in Abstract
1b	Structured summary of trial design, methods, results, and conclusions (for specific guidance see CONSORT for abstracts)	Abstract	Present: structured abstract with Background, Methods, Results, Conclusion
**INTRODUCTION**			
2a	Scientific background and explanation of rationale	Introduction, pp. 1–4	Comprehensive; covers RCC epidemiology, NMDA receptor mechanism, preclinical sequence data
2b	Specific objectives or hypotheses	Introduction, last 3 paragraphs	Two explicit hypotheses stated (K→Mg superiority; Mg→K non-inferiority to placebo)
**METHODS**			
3a	Description of trial design (such as parallel, factorial) including allocation ratio	Section 2.1	Parallel, 9-arm factorial RCT; allocation ratio 1:1:1:1:1:1:1:1:1 (unequal in practice)
3b	Important changes to methods after trial commencement (such as eligibility criteria), with reasons	—	No changes reported; not applicable
4a	Eligibility criteria for participants	Section 2.2	Inclusion/exclusion criteria fully specified (ASA I–III, age ≥18, open radical nephrectomy)
4b	Settings and locations where the data were collected	Section 2.1	University Clinical Centre of Serbia, Belgrade; August 2017–January 2026
5	The interventions for each group with sufficient details to allow replication, including how and when they were actually administered	Sections 2.3, 2.5	Full details: doses (ketamine 0.2 mg/kg, $MgSO_4$ 15 mg/kg, placebo 0.9% NaCl), timing (Drug A post-induction, Drug B +10 min), all 9 sequences specified

Item	Checklist Item (CONSORT 2025)	Location in Manuscript	Remarks
6a	Completely defined pre-specified primary and secondary outcome measures, including how and when they were assessed	Section 2.8	NRS (0–10) and VRS pain at rest and on movement; morphine consumption; 14 time points over 48 h
6b	Any changes to trial outcomes after the trial commenced, with reasons	—	None reported
7a	How sample size was determined	Section 2.9	A priori calculation: MCID 1.5 NRS, SD 2.5, α=0.05, power 80%; minimum 17/group; enrolled 208 (range 17–37/group)
7b	When applicable, explanation of any interim analyses and stopping guidelines	—	Not reported; no interim analyses performed
8a	Method used to generate the random allocation sequence	Section 2.3	Computer-based random number generator with block randomization
8b	Type of randomisation; details of any restriction (such as blocking and block size)	Section 2.3	Block randomization; block size not specified
9	Mechanism used to implement the random allocation sequence (such as sequentially numbered containers), describing any steps taken to conceal the sequence until interventions were assigned	Section 2.3	Maintained by independent coordinator; identical syringes of equal volume prepared by anesthesia nurse not involved in patient care
10	Who generated the random allocation sequence, who enrolled participants, and who assigned participants to interventions	Section 2.3	Independent coordinator generated sequence; anesthesia nurse prepared solutions; attending anesthesiologist assigned interventions
11a	If done, who was blinded after assignment to interventions (for example, participants, care providers, outcome assessors) and how	Section 2.3	Double-blind: attending anesthesiologist, surgical team, and all postoperative assessors blinded; identical syringes used
11b	If relevant, description of the similarity of interventions	Section 2.3	All solutions colorless, transparent, in identical syringes of equal volume
12a	Statistical methods used to compare groups for primary and secondary outcomes	Section 2.9	Kruskal-Wallis (overall); Mann-Whitney U (pairwise); Chi-square/Fisher's exact (categorical); Spearman correlation
12b	Methods for additional analyses, such as subgroup analyses and adjusted analyses	Section 2.9	Exploratory pooled analysis (all $MgSO_4$ groups vs. no-Mg groups); Spearman correlations for predictors
**RESULTS**			
13a	For each group, the numbers of participants who were randomly assigned, received intended treatment, and were analysed for the primary outcome	Section 3.1, Table 1	208 enrolled; group sizes: K→Mg n=32, Mg→K n=19, Pl→Pl n=37, others 17–24; all analysed (per protocol)

Item	Checklist Item (CONSORT 2025)	Location in Manuscript	Remarks
13b	For each group, losses and exclusions after randomisation, together with reasons	Section 3.1	No losses or exclusions after randomisation reported; all 208 analysed
14a	Dates defining the periods of recruitment and follow-up	Section 2.1	Recruitment: August 2017–January 2026; follow-up: 48 hours post-surgery
14b	Why the trial ended or was stopped	—	Trial completed as planned; no stopping rule invoked
15	A table showing baseline demographic and clinical characteristics for each group	Table 1	Table 1: age, weight, BMI, surgery duration, fentanyl dose, sex, smoking status — all groups
16	For each group, number of participants (denominator) included in each analysis and whether the analysis was by original assigned groups	Tables 2–4	Per-group n reported for all analyses; intention-to-treat (all 208 randomised patients)
17a	For each primary and secondary outcome, results for each group, and the estimated effect size and its precision (such as 95% confidence interval)	Tables 2, 3, 4	Median NRS (IQR) and p-values reported; CIs not reported — consider adding for key comparisons
17b	For binary outcomes, presentation of both absolute and relative effect sizes is recommended	—	Primary outcomes are continuous (NRS); binary outcomes (hallucinations, vomiting) reported as frequencies
18	Results of any other analyses performed, including subgroup analyses and adjusted analyses, distinguishing pre-specified from exploratory	Section 2.9, Discussion	Exploratory pooled Mg analysis; Spearman correlations; NRS–VRS convergence — labelled exploratory
19	All important harms or unintended effects in each group (for specific guidance see CONSORT for harms)	Section 3.4, Discussion	Zero hallucinations/psychomimetic events; sedation 5–9%; nausea/vomiting reported
**DISCUSSION**			
20	Trial limitations, addressing sources of potential bias, imprecision, and, if relevant, multiplicity of analyses	Discussion, Limitations paragraphs	Five limitations explicitly acknowledged (sample size, bolus-only protocol, CADSS not used, single centre, no chronic pain follow-up)
21	Generalisability (external validity, applicability) of the trial findings	Discussion, Limitations; Conclusion	Addressed: single centre, ASA I–III, open nephrectomy only; laparoscopic generalisability noted as future work
22	Interpretation consistent with results, balancing benefits and harms, and considering other relevant evidence	Discussion, Conclusion	Comprehensive; integrates preclinical data, existing RCTs, and safety profile; addresses morphine paradox
**OTHER INFORMATION**			
23	Registration number and name of trial registry	Abstract, Materials and Methods - section 2.1 (Study Design and Setting)	Trial registration number (ISRCTN83633282, ISRCTN registry) has

Item	Checklist Item (CONSORT 2025)	Location in Manuscript	Remarks
			been added to the Abstract and to section 2.1 (Study Design and Setting).
24	Where the full trial protocol can be accessed, if available	—	Not stated
25	Sources of funding and other support (such as supply of drugs), role of funders	Funding section	Funding section present but blank — please complete

** CONSORT 2025. Schulz KF, Moher D, Altman DG et al. CONSORT 2025 statement. BMJ. 2025.*

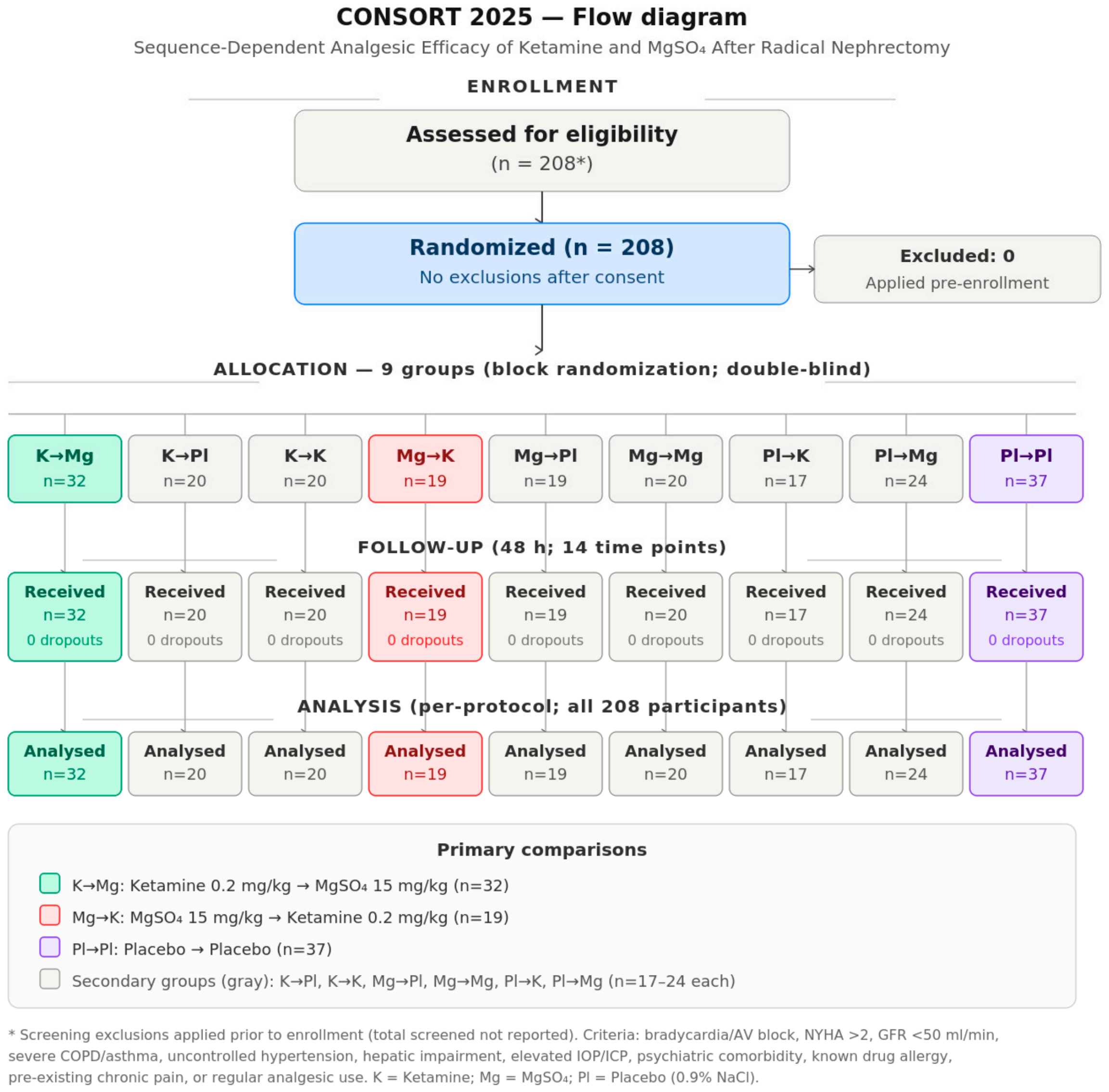

Figure S1. CONSORT flow diagram illustrating the enrollment, randomization, and analysis of participants across all nine treatment arms. Drug A was administered immediately after induction of anesthesia; Drug B was administered 10 minutes later. K, ketamine (0.2 mg/kg); Mg, magnesium sulfate (15 mg/kg); Pl, placebo (0.9% NaCl). All 208 randomized patients were included in the intention-to-treat analysis; there were no withdrawals or exclusions after randomization.
